# Postoperative Adjuvant Radiotherapy in Atypical Meningioma Patients: A Meta-Analysis Study

**DOI:** 10.3389/fonc.2021.787962

**Published:** 2021-12-02

**Authors:** Dengpan Song, Dingkang Xu, Hongjie Han, Qiang Gao, Mingchu Zhang, Fang Wang, Guoqing Wang, Fuyou Guo

**Affiliations:** ^1^ Department of Neurosurgery, The First Affiliated Hospital of Zhengzhou University, Zhengzhou, China; ^2^ Department of Neurosurgery, Pingdingshan Second People’s Hospital, Pingdingshan, China

**Keywords:** atypical meningioma, adjuvant radiotherapy, meta-analysis, gross total resection, subtotal resection, progression-free survival, overall survival

## Abstract

**Background and Purpose:**

Consensus regarding the need for adjuvant radiotherapy (RT) in patients with atypical meningiomas (AMs) is lacking. We compared the effects of adjuvant RT after surgery, gross total resection (GTR), and subtotal resection (STR) on progression-free survival (PFS) and overall survival (OS) in patients with AMs, respectively.

**Methods:**

We performed a systematic review and meta-analysis of the literature published in PubMed, Embase, and the Cochrane Library from inception to February 1, 2021, to identify articles comparing the PFS and OS of patients receiving postoperative RT after surgery, GTR and STR.

**Results:**

We identified 2307 unique studies; 24 articles including 3078 patients met the inclusion criteria. The sensitivity analysis results showed that for patients undergoing undifferentiated surgical resection, adjuvant RT reduced tumor recurrence (HR=0.70, p<0.0001) with no significant effect on survival (HR=0.89, p=0.49). Postoperative RT significantly increased PFS (HR=0.69, p=0.01) and OS (HR=0.55, p=0.007) in patients undergoing GTR. The same improvement was observed in patients undergoing STR plus RT (PFS: HR=0.41, p<0.00001; OS: HR=0.47, p=0.01). A subgroup analysis of RT in patients undergoing GTR showed no change in PFS in patients undergoing Simpson grade I and II resection (HR=1.82, p=0.22) but significant improvement in patients undergoing Simpson grade III resection (HR=0.64, p=0.02).

**Conclusion:**

Regardless of whether GTR or STR was performed, postoperative RT improved PFS and OS to varying degrees. Especially for patients undergoing Simpson grade III or IV resection, postoperative RT confers the benefits for recurrence and survival.

## Introduction

Meningiomas are the most common primary tumors of the brain, representing more than one-third of all intracranial tumors (1). According to the most recent WHO definition, meningioma should be classified according to 3 histological grades, with benign meningioma (BM) classified as grade I, atypical meningioma (AM) as grade II, and malignant meningioma (MM) as grade III (2). Atypical meningiomas (AMs) accounts for about 15-20% of all meningiomas, and associated with a higher risk of recurrence and a worse prognosis than benign meningiomas (3, 4). Maximal safe surgical resection is currently the preferred treatment for atypical meningiomas, but there is no clear consensus on the use of adjuvant radiotherapy (RT) in these patients (5). There are two points to consider, namely, whether adjuvant radiation therapy can significantly improve the patient’s prognosis and whether the side effects of adjuvant radiation therapy can offset the benefits (2, 6).

Although the effect of postoperative RT on AMs has been analyzed in many reports, the results were inconsistent. Therefore, it is necessary to perform a meta-analysis to evaluate the efficacy of surgical resection with RT on survival outcomes, including overall survival (OS) and progression-free survival (PFS), in patients suffering from AMs.

## Methods

### Search Strategy

We comprehensively searched eligible studies using several electronic databases, including the PubMed, Embase, and Cochrane databases and followed PRISMA guidelines. Search terms included a strategic combination of ‘atypical’ AND ‘meningioma’, or ‘atypical meningioma’ or ‘grade II meningioma’. All papers published until February 1, 2021 were included. The titles and abstracts of each article searched were reviewed to exclude any apparently unrelated research. The full texts of the remaining articles were read to determine whether they contained information on the subject under review.

### Study Selection

Two investigators independently reviewed each eligible study, with a consensus being reached by the third investigator when there was a disagreement between the two investigators. Articles that satisfied the following criteria were included: (1) cohort studies or randomized controlled trials, (2) patients with atypical meningioma verified by pathology, (3) studies that investigated different treatment modalities, including GTR and STR plus RT, and (4) OS and/or PFS data that were provided or allowed for the calculation of hazard ratios (HRs) with corresponding 95% confidence intervals (CIs). Studies were excluded based on any of the following criteria: (1) reviews, letters, case reports, and database-based studies; (2) studies with a sample size of less than 20; (3) non-English studies, studies with duplicate data; and (4) studies that lacked key information for calculation. The definitions of GTR and STR were based on the description in the original article. In general, GTR was defined as a Simpson Grade I or II tumor resection, or Simpson Grade I, II or III tumor resection, and STR was defined as a Simpson Grade IV tumor resection. In addition, to further clarify the potential impact of GTR plus RT on AM under real-world conditions, a subgroup analysis of the GTR group was performed according to Simpson’s classification. Radiotherapy was considered to include both conventional radiotherapy and stereotactic radiosurgery (SRS).

### Quality Assessment

Two investigators independently assessed the quality of each eligible study using the Newcastle-Ottawa Scale (7). Three aspects were generally assessed: population selection, study comparability, and reporting of the outcome, with a score ranging from 0 to 9. Studies with a score greater than six were considered to be of high quality.

### Statistical Analysis

All the comparisons were based on data from cohort studies. The endpoints of interest in the analyses were OS and PFS. A hazard ratio (HR) with a 95% CI was used to evaluate the association of postoperative radiotherapy. The lnHRs were considered to obey a normal distribution. We extracted the HRs and corresponding 95% CIs of the multivariate analysis explicitly given in these articles; otherwise, the HRs and 95% CIs of the univariate analysis were utilized. If the above value was not provided in the paper, the Kaplan-Meier survival curve in the paper was used to transform the Figure into a data sheet and used the log rank test to obtain the lnHR, 95% CI and SE (8). The I^2^ statistic and Cochrane Q test were used to analyze between-study heterogeneity (9). Data analyses were performed using Review Manager software version 5.3 (The Cochrane Collaboration Oxford, United Kingdom). When I^2^ < 50% or a P value > 0.10 was identified, indicating homogeneity among studies, we used the fixed-effects model; otherwise, a random-effects model was adopted. Publication bias was determined using the funnel graph. We performed a sensitivity analysis by omitting each study in turn. A P value of <0.05 indicated a statistically significant difference.

## Results

### Literature Selection, Quality Evaluation, and Demographics

**Figure 1:**
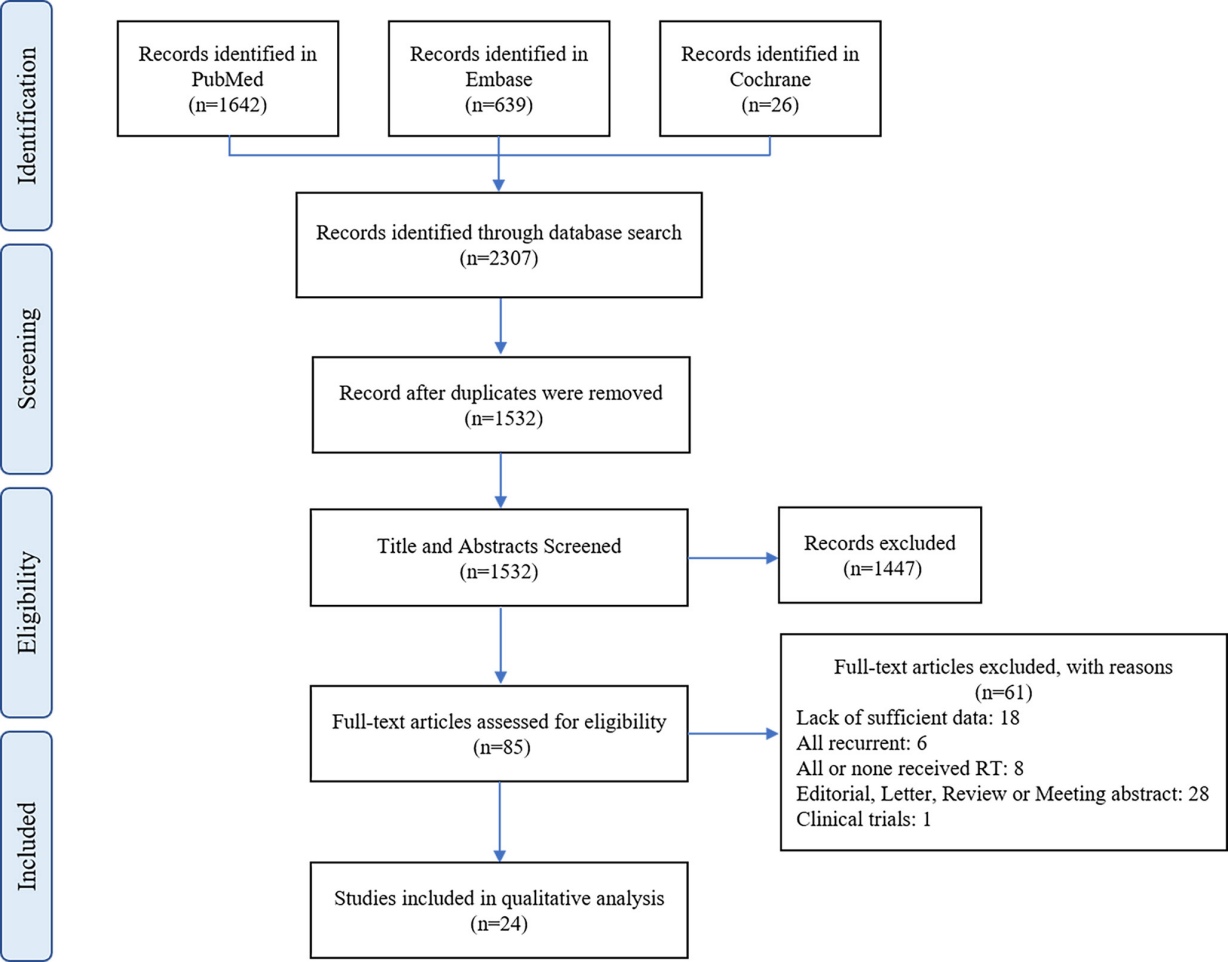
The flow diagram of the selection process as per PRISMA.

The process of literature screening based on the inclusion and exclusion criteria is depicted in **Figure 1**. The initial search yielded 2307 results. Of these, 775 studies were excluded because they were duplicates. After scanning the titles and abstracts, 85 studies were retained for further analysis. Finally, after a full-text screening, a total of 24 were eligible for inclusion in the meta-analysis. The quality of 24 comparative studies with a total of 3078 patients is summarized in **Supplementary Table 1**, and the results of our systematic analysis of patients with AMs undergoing postoperative RT are detailed in **Table 1**. The mean age was 57.17 years, and the male to female ratio was 1:1.26. The mean RT dose was 56.42 Gy and mean follow-up was 55.7 months.

**Table 1:** Demographics.

Author & year	Study duration (standard)	Design	Country	Sample size	Male: Female	Median age	Median follow-up (months)	Median dose (Gy)	HR					
									Surgery+RT *vs* Surgery		GTR+RT *vs* GTR		STR+RT *vs* STR	
									PFS	OS	PFS	OS	PFS	OS
Jo (10)	1997-2008 (2000)	Retrospective	Korea	35	18:17	40	56	NR	0.626	NR	NR	NR	0.204	NR
Mair (11)	2001-2010 (2000)	Retrospective	UK	114	55:59	57	NR	51.8	0.831	NR	NR	NR	NR	NR
Komotar (12)	1992-2011 (2000)	Retrospective	US	45	20:25	56.1	44.1	59.4	NR	NR	0.236	NR	NR	NR
Hammouche (13)	1996-2009 (2007)	Retrospective	UK	79	43:36	58	50	56.2	0.960	NR	NR	NR	NR	NR
Aizer (14)	1997-2011 (NR)	Retrospective	US	91	41:50	57	58.8	60	0.240	0.210	0.250	0.247	NR	NR
Wang (15)	2001-2009 (2007)	Retrospective	China	28	13:15	56.8	57.4	57	NR	NR	0.029	0.354	NR	NR
Zhao (16)	2001-2011 (2000)	Retrospective	China	89	42:47	53.3	25	NR	0.722	1.111	NR	NR	NR	NR
Champeaux (17)	2007-2015 (2007)	Retrospective	UK	194	93:101	54.2	52.8	NR	3.820	1.050	NR	NR	NR	NR
Jenkinson (18)	2001-2010 (2007)	Retrospective	UK, Italy, Ireland,	133	68:65	62	57.4	60	NR	NR	0.842	0.926	NR	NR
Endo (19)	2000-2013 (2007)	Retrospective	Japan	45	25:20	58.7	81	50	1.200	NR	NR	NR	NR	NR
Bagshaw (20)	1991-2014 (2007)	Retrospective	US	63	29:33	53	42	54	0.388	NR	0.026	NR	NR	NR
Graffeo (21)	1988-2011 (2016)	Retrospective	US	69	25:44	60	95	54	NR	NR	1.781	0.492	NR	NR
Phonwijit (22)	2004-2014 (2007)	Retrospective	Thailand	126	42:84	55	52	NR	0.402	NR	NR	NR	NR	NR
Dohm (23)	1993-2014 (2007)	Retrospective	US	83	32:51	63.6	36.9	55.7	0.430	0.523	0.657	NR	0.193	NR
Masalha (24)	2001-2015 (2016)	Retrospective	Germany	161	76:85	70	62.4	NR	0.860	NR	NR	NR	NR	NR
Shakir (25)	1992-2013 (2007)	Retrospective	Canada	70	32:38	62	67	54	0.046	NR	0.017	NR	0.081	NR
Chen (26)	1993-2014 (2000/2007)	Retrospective	US	182	71:111	57	52.8	59.4	0.150	NR	0.010	0.494	0.180	0.642
Li (27)	2008-2015 (2007)	Retrospective	China	302	136:166	51	41.6	NR	0.662	0.096	0.811	0.036	0.470	0.401
Zhu (28)	2005-2008 (2000)	Retrospective	China	99	48:51	NR	76.5	NR	NR	NR	0.695	0.646	0.238	0.223
Streckert (4)	1991-2018 (2016)	Retrospective	Germany	138	74:64	62	62	59.4	3.409	NR	4.340	NR	1.670	NR
Wang (29)	2009-2018 (2007/2016)	Retrospective	China	263	99:164	52	41	56	0.629	0.026	0.966	0.026	0.246	NR
Keric (30)	2007-2017 (2007)	Retrospective	Germany	258	117:141	60	31	NR	0.788	NR	2.776	NR	0.724	NR
Lee (5)	2000-2015 (2000/2007)	Retrospective	US	230	93:137	56.6	82.8	59.4	0.210	0.987	0.451	NR	0.471	NR
Garcia-Segura (31)	1995-2015 (2007)	Retrospective	US	181	72:109	59.6	NR	NR	4.352	NR	6.328	NR	1.793	NR

NR, no reported.

### Results of the Meta-Analysis

#### Meta-Analysis of PFS and OS Between Surgery+RT and Surgery

**Figure 2:**
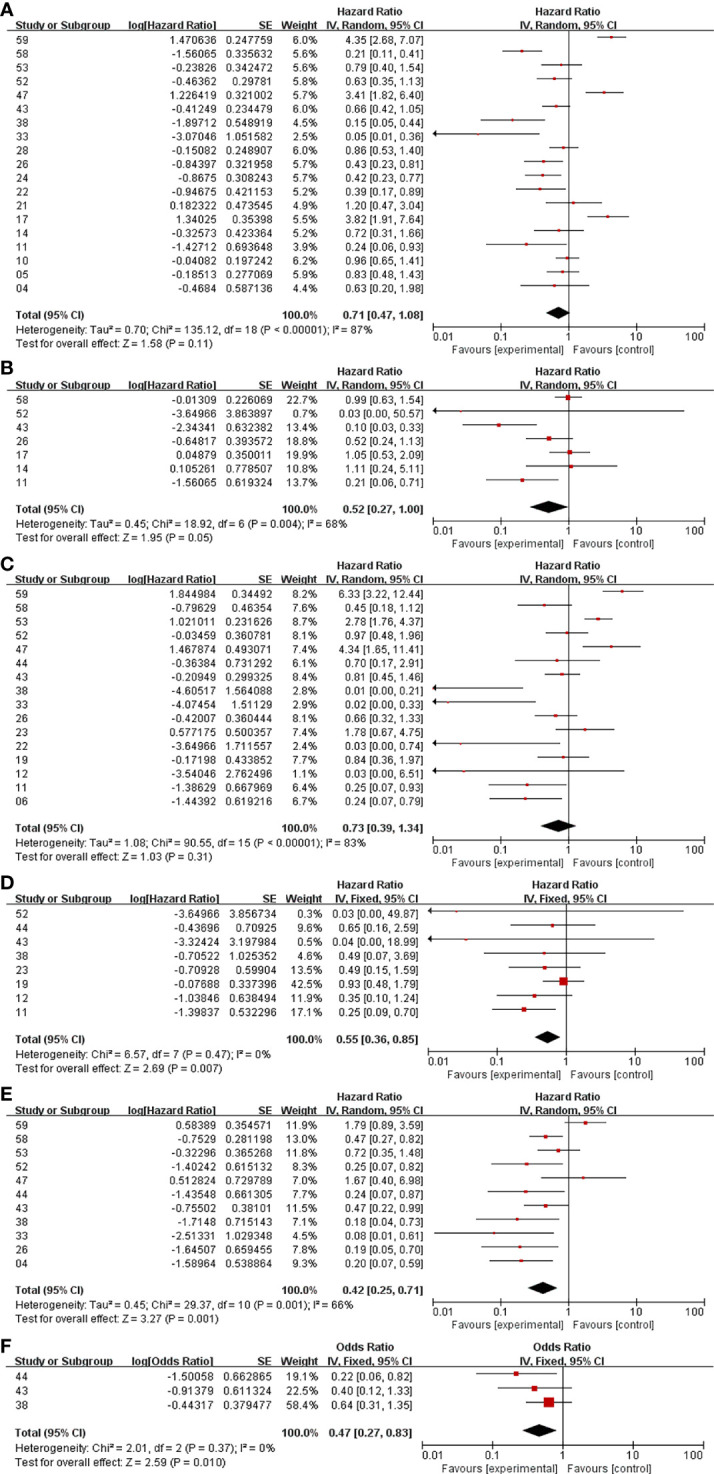
Meta-analysis. Surgery+RT *vs* Surgery, PFS **(A)**. Surgery+RT *vs* Surgery, OS **(B)**. GTR+RT *vs* GTR, PFS **(C)**. GTR+RT *vs* GTR, OS **(D)**. STR+RT *vs* STR, PFS **(E)**. STR+RT *vs* STR, OS **(F)**.

**Figure 3:**
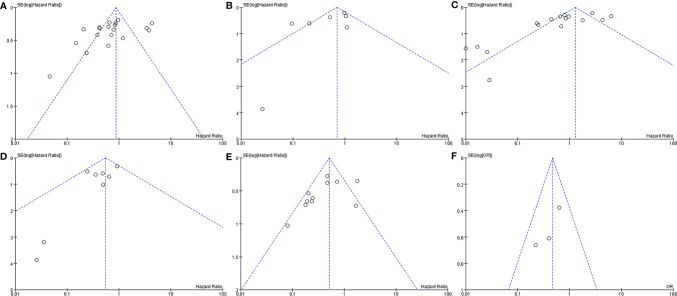
Funnel plot. Surgery+RT vs Surgery, PFS **(A)**. Surgery+RT vs Surgery, OS **(B)**. GTR+RT vs GTR, PFS **(C)**. GTR+RT vs GTR, OS **(D)**. STR+RT vs STR, PFS **(E)**. STR+RT vs STR, OS **(F)**.

**Figure 4:**
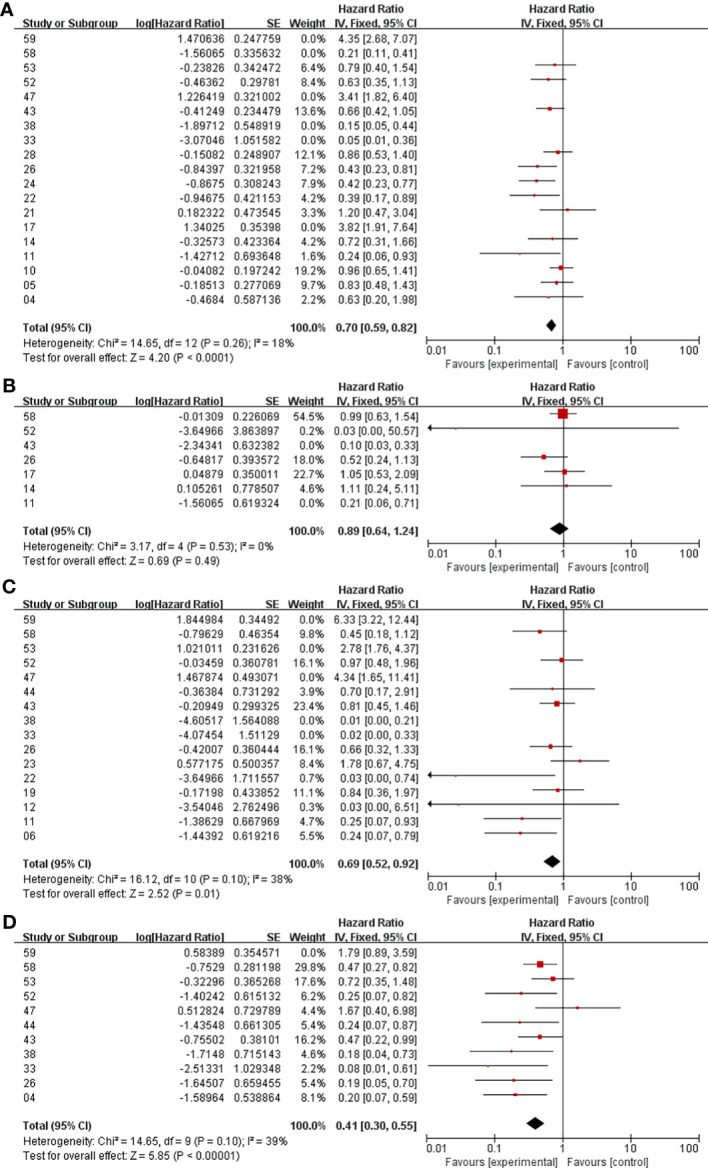
Meta-analysis after sensitivity analysis. Surgery+RT vs Surgery, PFS **(A)**. Surgery+RT vs Surgery, OS **(B)**. GTR+RT vs GTR, PFS **(C)**. STR+RT vs STR, PFS **(D)**.

**Figure 5:**
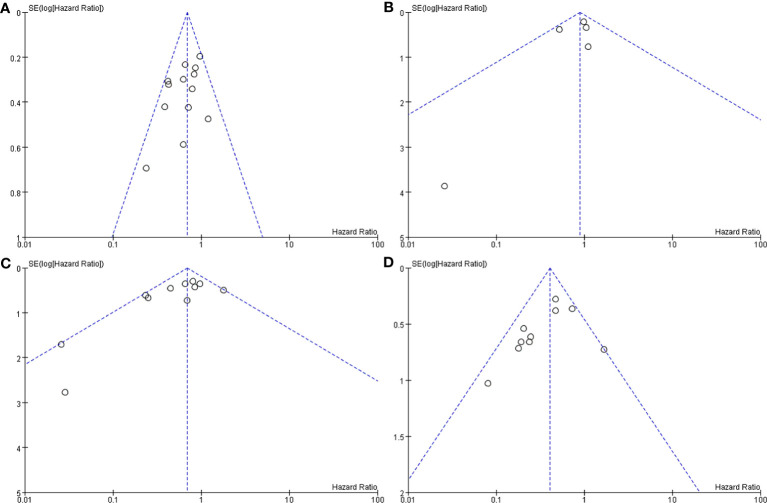
Funnel plot after sensitivity analysis. Surgery+RT vs Surgery, PFS **(A)**. Surgery+RT vs Surgery, OS **(B)**. GTR+RT vs GTR, PFS **(C)**. STR+RT vs STR, PFS **(D)**.

In the analysis of PFS and OS in atypical meningioma patients treated with surgery and RT or surgery alone, 19 and 7 studies were included, respectively, and the results are shown in **Figure 2**. For PFS, the P value of the Q statistic and the Higgins I^2^ statistic for heterogeneity were 0.00001 and 87%, respectively. Similarly, for OS, the P value and I^2^ were 0.004 and 68%, respectively. This result indicated that both studies were heterogeneous, so we applied a random-effects model. Benefits of RT were found in both the PFS and OS analyses (PFS: HR = 0.71, 95% CI: 0.47-1.08, P = 0.11, and OS: HR = 0.52, 95% CI: 0.27-1.00, P = 0.05). The publication bias of the PFS and OS analyses is shown as a funnel graph in **Figure 3**. The results show that both of them have significant publication bias. A significant improvement in publication bias was observed after 6 and 2 articles were removed by the sensitivity analysis (**Figure 4**). The Higgins I^2^ statistics for heterogeneity were 18% (p = 0.26) and 0% (p = 0.53), respectively, which indicates that the remaining studies are homogeneous. Thus, the fixed-effect model is used to present the results in **Figure 5**. The improve of adjuvant RT on prognosis remained (PFS: HR = 0.70, 95% CI: 0.59-0.82, P < 0.0001, and OS: HR = 0.89,95% CI: 0.64-1.24, P = 0.49).

#### Meta-Analysis of PFS and OS Between GTR With RT and GTR

In the analyses of PFS and OS in AM patients treated with GTR+RT and GTR, 16 and 8 studies were included, respectively, which are shown in **Figure 2**. In the PFS analysis, the P value of the Q statistic and the Higgins I^2^ statistic for heterogeneity were 0.00001 and 83%, respectively. In the OS analysis, the P value and I^2^ were 0.47 and 0%, respectively. This result indicated that the former was heterogeneous and the latter was homoplasmic, so we applied a random-effects model and fixed-effects model, respectively. Benefits of RT were found in both the PFS and OS analyses (PFS: HR = 0.73, 95% CI: 0.39-1.34, P = 0.31, and OS: HR = 0.55, 95% CI: 0.36-0.85, P = 0.007). As shown in the funnel diagram in **Figure 3**, significant publication bias was observed in the PFS results. After the removal of 5 studies, the bias was improved (I^2^ = 38%, P = 0.01), and a fixed-effects model was used to present the results (**Figures 4**, **5**). The results of the sensitivity analysis indicated that adjuvant RT also significantly reduced the recurrence rate in patients with GTR (PFS: HR = 0.69, 95% CI: 0.52-0.92, P = 0.01).

#### Meta-Analysis of PFS and OS Between STR With RT and STR Alone

In the analyses of PFS and OS in AM patients treated with STR+RT and STR alone, 11 and 3 studies were included, respectively, and the results are shown in **Figure 2**. In the PFS analysis, the P value of the Q statistic and the Higgins I^2^ statistic for heterogeneity were 0.001 and 66%, respectively. This implies the existence of heterogeneity, so we applied a random-effects model. In contrast, the fixed-effect model was chosen for the OS analysis because of homogeneity (I^2^ = 0% and p=0.37). Benefits of RT were found in both the PFS and OS analyses (PFS: HR = 0.42, 95% CI: 0.25-0.71, P = 0.001, and OS: R = 0.47, 95% CI: 0.27-0.83, P = 0.01). A funnel plot (**Figure 3**) confirmed the existence of publication bias in the PFS results, which was significantly improved after Garcia-Segura’s study (31) were removed by the sensitivity analysis (I^2^ = 39%, p=0.1), so a fixed-effects model was used to present the results (**Figures 4**, **5**). The PFS was still significantly improved after the sensitivity analysis (PFS: HR = 0.41, 95% CI: 0.30-0.55, P < 0.00001).

## Discussion

According to the latest EANO guidelines, maximum surgical resection with guaranteed safety is currently recognized as the preferred treatment for atypical meningioma (32). However, to date, there are no clear conclusions regarding whether postoperative radiotherapy is needed in patients with AMs (32). We performed the largest systematic review to date and extracted Hazard Ratio (HR) data with higher evidence level to compare the impact of STR and GTR on OS and/or PFS in AM patients with a rigorous assessment of the quality of the existing evidence. At the same time, we are the first to perform a subgroup analysis of different Simpson excision grade methods for GTR. Therefore, our results and conclusions have higher reference value.

### Surgery With RT and Surgery Alone

Much of the literature does not provide detailed data on GTR and STR but rather combines them into a single analysis. We are the first study to perform a meta-analysis with these data. Because of the large amount of related literature and a large sample size, this part of the analysis also has some value. Of the 19 articles including PFS, 15 reported HR < 1 for RT, of which 7 showed significant statistical significance, while only 4 reported the opposite results. Similarly, 5 of 7 studies examined the positive effects of RT on OS. After the sensitivity analysis, postoperative RT was associated with a 30% reduction in recurrence (p<0.0001) and an 11% reduction in mortality (p=0.49) compared with surgery alone, especially for the former, indicating that postoperative RT was associated with a significant improvement in PFS. Therefore, it is considered reasonable to consider postoperative RT for patients when the extent of surgical resection cannot be determined.

### GTR With RT and GTR Alone

**Figure 6:**
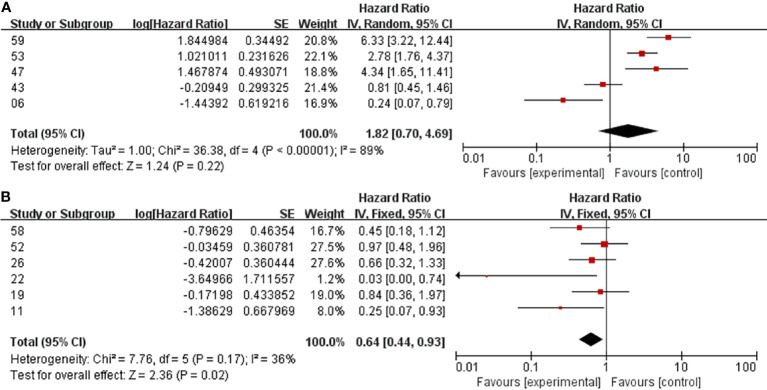
Subgroup analysis. GTR+RT vs GTR, PFS, GTR= Simpson Grade I or II tumor resection **(A)**. GTR+RT vs GTR, PFS, GTR= Simpson Grade I, II or III tumor resection **(B)**.

The debate over whether postoperative RT should be routinely performed in patients with GTR is most intense. Some people suggest that because of the thoroughness of resection, patients with GTR have fewer tumor recurrence events and longer survival times than patients with STR, so no postoperative RT is required (11, 29). A meta-analysis by Hasan et al. (33) focused specifically on the potential benefits of adjuvant RT after the complete removal of atypical meningiomas, with no clear benefits reported in terms of local control or 5-year survival. Even in Garcia-Segura’s cohort, adjuvant RT was associated with worse PFS and OS (31). However, in a prospective phase II study involving 15 centers in seven countries (34), as the highest level of inclusion in the literature, the data showed that the 3-year PFS for AM patients undergoing complete resection was greater than 70% when treated with high-dose (60 Gy) RT. In our analysis, postoperative RT was negatively correlated with recurrence and mortality across all the literature. For OS, the HR after GTR + RT in all studies was < 1, but for PFS, the heterogeneity among the articles was greater. The heterogeneity may be due to differences in the definitions of GTR and STR and differences in treatment protocols or techniques in different studies. After the deletion of 5 articles in the sensitivity analysis, there was less residual heterogeneity. Studies have shown that RT after GTR could significantly reduce the rate of recurrence. As one of the highlights, we are the first to perform a subgroup analysis of GTR. Five of these studies defined GTR as Simpson grade I or II tumor resection (**Figure 6**), while six studies included grade III resection. There was no significant effect on the recurrence rate in patients with RT after GTR defined as Simpson grade I or II tumor resection (HR=1.82, p=0.22), while PFS may be significantly prolonged with postoperative adjuvant RT in GTR, including grade III resection (HR=0.64, p=0.02). However, the findings related to the former should be interpreted with caution because of the small sample size, high heterogeneity (I^2^ = 89%) and lack of statistical significance (p=0.22). We suggest that grade III resection should not be attributed to GTR but should be treated as STR based on a combination of surgical records and postoperative MRI examination. According to our clinical experience, tumors in sites such as the cavernous sinus, the paraclinoid process, and the petroclival region may adhere to important structures such as the internal carotid artery, the basilar artery, and the brain stem. Although postoperative MRI and other imaging studies have failed to detect residual tumors, the presence of residual parenchyma is noted in the surgical record, and postoperative RT is recommended, especially in view of OS improvement. In summary, we believe that for patients with GTR, postoperative RT should be given appropriately, but tumor recurrence should be closely monitored, especially in Simpson grade I or II resection patients. In addition, the ROAM/EORTC 1308 trial (ISRCTN71502099), a multicenter, phase III, randomized controlled trial, has been developed to better answer whether early adjuvant radiotherapy for patients who have undergone GTR of AMs reduces recurrences compared with monitoring (35).

### STR With RT and STR Alone

It is generally accepted that patients with STR should be treated with postoperative radiation due to residual tumors after surgery (26), especially for PFS benefits (36). In Pant’s study cohort, 97% of patients who received radiation immediately after the initial resection had a recurrence rate, compared with 15% of patients who did not receive radiation (36). However, in Streckert’s study, none of the analyzed radiological features were correlated with survival (4). Garcia-Segura et al. even found that STR with RT significantly predicted tumor recurrence (31). Our results after sensitivity analysis confirmed that STR with postoperative RT reduced recurrence by 59% and mortality by 53%, both of which were statistically significant. To this end, we recommend that all STR patients undergo postoperative RT under appropriate conditions to extend PFS and OS.

### Radiotherapy Toxicity

There may be some side effects from radiotherapy, which must be considered for postoperative RT (37). The Common Terminology Criteria for Adverse Effects grading scale (version 4) has been used to observe and describe toxicity from RT and is usually classified into 4 levels. Levels 1 and 2 are more common, while level 4 is extremely rare. The relevant statistics are shown in **Table 2**. In Shakir’s study, grade 1 or 2 toxicities were noted in 8 patients (radiotherapy-attributed toxicity rate was 20%) and included headache (4 patients), dizziness (3 patients) and paresthesia (1 patient). These toxicities were self-limiting and managed with short-course corticotherapy (25). In Chen’s and Dohma’s study cohorts, there were 5 (12%) and 8 (15%) cases with grade 2+ adverse effects of RT, respectively, and 1 case with grade 4 toxicity. The former suffered from cerebral hemorrhage and died, while the latter developed medically intractable epileptic seizures and had to be hospitalized (23, 26). According to our analysis, we suggest that although the possible side effects are not negligible, there are overall benefits to postoperative RT relative to significant improvements in recurrence and survival. Close observation, follow-up and evaluation of adverse reactions to real-time adjustment of regimens, and active symptomatic treatment should be performed in conjunction with postoperative RT.

**Table 2:** Toxicity of postoperative adjuvant RT.

Study	RT Toxicity (%)	Description
Bagshaw et al. (20)	14	Grade 2 or 3 toxicities
Graffeo et al. (21)	12.5	
Dohm et al. (23)	15	Grade 3 or 4 toxicities (radiation necrosis, cognitive disturbances, peripheral neuropathy, seizures, aphasia, optic nerve disorders)
Shakri et al. (25)	20	Grade 1 or 2 toxicities (headache, dizziness, aparesthesia)
Chen et al. (26)	14	Grade 2+ toxicities (radiation necrosis, lower-extremity paresis, short-term visual blurring, transient radiation-induced encephalopathy) Intracranial hemorrhage

### Assessment of Heterogeneity

There is significant clinical and methodological heterogeneity in our systematic review, which can be evidenced by the wide range of patient numbers, ages, follow-up times, radiation doses, modes of radiotherapy and definitions of GTR and STR classifications. The only known prognostic factor for AM is the extent of resection; however, age (38), tumor volume, and Mib-1 (29) have been associated with PFS and OS in a number of single institution studies. With regard to some of the more heterogeneous results we have obtained, we suspect that the possible reasons are the different definitions of GTR and STR in different studies, and the application time and methods of radiation therapy after surgery were inconsistent. In summary, we used a random-effects model for heterogeneity > 50 and carried out sensitivity analyses and subsection analyses.

### Limitations

The WHO classification definition of AMs changed in 2000, 2007 and 2016. All the studies included were retrospective cohort studies. Therefore, more prospective and long-term follow-up studies are needed to better verify the impact of RT on prognosis. The large sample size also brings some heterogeneity. Finally, the exclusion of non-English literature may leading to potential language bias.

## Conclusion

Maximum surgical resection with guaranteed safety is currently recognized as the preferred treatment for AM, but whether to perform postoperative RT remains a controversial issue. To the best of our knowledge, the present study is the largest meta-analysis on this topic using high-evidence-level HR data and reveals the benefits of postoperative RT assistance in patients with AMs, especially for OS. Regardless of whether GTR or STR is performed, postoperative RT was found to effectively increase PFS and OS to varying degrees. Especially for patients undergoing Simpson grade III or IV resection, postoperative RT confers the benefits for recurrence and survival. Moreover, long-term surveillance should be tailored based on the Simpson grade of AMs. Clinical trials such as ROAM will investigate further.

## Data Availability Statement

The original contributions presented in the study are included in the article/**Supplementary Material**. Further inquiries can be directed to the corresponding author.

## Author Contributions

DS and DX have contributed equally. DS: data processing and manuscript writing, DX: conception and data collection. HH and GW: critical review. QG, MZ, and FW: data collection and evaluation. FG: conception and critical review. All authors contributed to the article and approved the submitted version.

## Conflict of Interest

The authors declare that the research was conducted in the absence of any commercial or financial relationships that could be construed as a potential conflict of interest.

## Publisher’s Note

All claims expressed in this article are solely those of the authors and do not necessarily represent those of their affiliated organizations, or those of the publisher, the editors and the reviewers. Any product that may be evaluated in this article, or claim that may be made by its manufacturer, is not guaranteed or endorsed by the publisher.
