# Supplementary material for: Postoperative Adjuvant Radiotherapy in Atypical Meningioma Patients: A Meta-Analysis Study
Source: Front Oncol. 2021 Dec 2;11:787962. doi: 10.3389/fonc.2021.787962 (PMC8674463; doi:10.3389/fonc.2021.787962)
Supplement: Supplementary file 1 [file Table_1.docx]

**Supplementary Table 1** Quality assessment of cohort studies included in the meta-analysis according to the Newcastle-Ottawa Scale.

| Authors and year of publication | Representativeness of the exposed cohort | Selection of the unexposed cohort | Ascertainment of exposure | Demonstration that outcome of interest was not present at start of study | Comparability of cohorts on the basis of the design or analysis | Assessment of outcome | Follow-up was long enough for outcomes to occur | Adequacy of follow-up of cohorts | Overall quality score |
| --- | --- | --- | --- | --- | --- | --- | --- | --- | --- |
| Jo (2010) (10) | * | * | * |  | ** | * | * | * | 8 |
| Mair (2011) (11) | * | * | * |  | * | * | * | * | 7 |
| Komotar (2012) (12) | * | * | * |  | ** | * | * | * | 8 |
| Hammouche (2014) (13) | * | * | * |  | ** | * | * | * | 8 |
| Aizer (2014) (14) | * | * | * |  | ** | * | * | * | 8 |
| Wang (2014) (15) | * | * | * |  |  | * | * | * | 6 |
| Zhao (2014) (16) | * | * | * |  | * | * | * | * | 7 |
| Champeaux (2016) (17) | * | * | * |  |  | * | * | * | 6 |
| Jenkinson (2016) (18) | * | * | * |  |  | * | * | * | 6 |
| Endo (2016) (19) | * | * | * |  |  | * | * | * | 6 |
| Bagshaw (2016) (20) | * | * | * |  |  | * | * | * | 6 |
| Graffeo (2017) (21) | * | * | * |  | * | * | * | * | 7 |
| Phonwijit (2017) (22) | * | * | * |  | ** | * | * | * | 8 |
| Dohm (2017) (23) | * | * | * |  | * | * | * | * | 7 |
| Masalha (2017) (24) | * | * | * |  | ** | * | * | * | 8 |
| Shakir (2018) (25) | * | * | * |  | ** | * | * | * | 8 |
| Chen (2018) (26) | * | * | * |  | ** | * | * | * | 8 |
| Li (2018) (27) | * | * | * |  | ** | * | * | * | 8 |
| Zhu (2019) (28) | * | * | * |  | * | * | * | * | 7 |
| Streckert (2019) (4) | * | * | * |  | ** | * | * | * | 8 |
| Wang (2019) (29) | * | * | * |  | ** | * | * | * | 8 |
| Keric (2020) (30) | * | * | * |  |  | * | * | * | 6 |
| Lee (2020) (5) | * | * | * |  | * | * | * | * | 7 |
| Garcia-Segura (2020) (31) | * | * | * |  |  | * | * | * | 6 |
